# *FLT3*-ITD Measurable Residual Disease in Acute Myeloid Leukemia: Implications for *FLT3* Inhibitor-Based Therapies

**DOI:** 10.3390/cancers18162586

**Published:** 2026-08-11

**Authors:** Giorgia Silvestrini, Serena Travaglini, Luca Guarnera, Nicole Lelli, Mariadomenica Divona, Elisa Casciani, Sara Ceccolini, Giulia Falconi, Tiziana Ottone, Maria Teresa Voso

**Affiliations:** 1 Department of Biomedicine and Prevention, University of Rome Tor Vergata, 00133 Rome, Italy; giorgia.silvestrini@uniroma2.it (G.S.); serena.travaglini@uniroma2.it (S.T.); mariadomenica.divona@ptvonline.it (M.D.); elisa.casciani@ptvonline.it (E.C.); sara.ceccolini@ptvonline.it (S.C.); giulia.falconi@ptvonline.it (G.F.); tiziana.ottone@uniroma2.it (T.O.); 2 Department of Experimental Medicine, University of Rome Tor Vergata, 00133 Rome, Italy; 3 PhD Program in Immunology, Molecular Medicine and Applied Biotechnology, Department of Biomedicine and Prevention, University of Rome Tor Vergata, 00133 Rome, Italy; luca.guarnera@uniroma2.it (L.G.); nicole.lelli@students.uniroma2.eu (N.L.); 4 Saint Camillus International University of Health Sciences, 00131 Rome, Italy; 5 Neuro-Oncohematology Unit, Santa Lucia Foundation, Istituto di Ricovero e Cura a Carattere Scientifico (I.R.C.C.S.), 00143 Rome, Italy

**Keywords:** acute myeloid leukemia, *FLT3*-ITD, measurable residual disease, MRD, next-generation sequencing, NGS, allogeneic stem-cell transplantation, allo-HSCT, FLT3 inhibitors, FLT3i

## Abstract

*Fms-related receptor tyrosine kinase 3* internal tandem duplication (*FLT3*-ITD) mutations occur in approximately 20–25% of patients with acute myeloid leukemia (AML) and are associated with increased relapse risk and inferior survival outcomes. Although measurable residual disease (MRD) has become a key prognostic tool for guiding post-remission treatment decisions, *FLT3*-ITD was historically considered a suboptimal MRD marker because of its subclonal nature, structural heterogeneity and dynamic behavior during disease evolution. In addition, *FLT3*-ITD has not yet been fully integrated into routine MRD monitoring due to methodological limitations and a lack of standardized workflows. The latest European LeukemiaNet (ELN)-DAVID 2025 recommendations stressed the use of ultra-high sensitivity (UHS) next-generation sequencing (NGS) technologies to detect *FLT3*-ITD MRD with improved precision, enabling reliable longitudinal tracking of patient-specific clones at very low variant allele frequencies (VAF). Indeed, despite prospective evidence supporting this approach remaining limited, *FLT3*-ITD-based MRD monitoring is emerging as a clinically relevant prognostic indicator, contributing to the identification of patients at increased risk of relapse and refining risk stratification, while also informing therapeutic decision-making, particularly in the peri-transplant setting. The present review summarizes the biological underpinnings of *FLT3*-ITD mutated (*FLT3*-ITD^mut^) AML, discusses the methodological challenges of MRD detection, and critically evaluates the evolving role of MRD in refining relapse prediction, supporting post-remission therapy tailoring, and contributing to a harmonized framework for *FLT3*-ITD^mut^ AML management.

## Simple Summary

Acute myeloid leukemia (AML) is a clinically and molecularly heterogeneous disease, driven by a plethora of genetic abnormalities, among which *FLT3* mutations are among the most common in adult patients. Given the association between *FLT3*-ITD mutations and the increased risk of relapse, the study of these mutations in the context of measurable residual disease (MRD) plays a crucial role in the clinical management of AML. Of note, the advent of next-generation sequencing (NGS) techniques has improved mutation detection and enabled sequence-level characterization of ITDs. In this review, we aim to summarize and discuss the importance of *FLT3*-ITD NGS-based MRD as a new informative tool potentially guiding transplant- and post-remission-oriented decision-making in *FLT3*-ITD^mut^ AML.

## 1. Introduction

Acute myeloid leukemia (AML) is a clonal hematopoietic neoplasm characterized by a broad spectrum of genetic alterations that drive classification, risk stratification and therapeutic decisions [1,2,3]. This plasticity of the mutational pattern of AML is the result of a multistep process of clonal evolution in which a founder mutation—most commonly affecting genes encoding epigenetic regulators—can acquire additional abnormalities that accumulate over time [4,5]. In particular, the acquisition of leukemia-defining lesions, including *NPM1* (*nucleophosmin 1*) [6] mutations and activating signaling pathway alterations, has been shown to drive overt leukemic transformation. An outstanding example of clonal evolution is represented by *fms-related receptor tyrosine kinase 3* internal tandem duplication (*FLT3*-ITD) mutations [7,8], which, especially following FLT3 inhibitor (FLT3i) therapy, may become undetectable at relapse, with the acquisition of additional mutations accountable for therapy resistance [9]. Internal tandem duplications (ITDs) in the *FLT3* gene, affecting the juxtamembrane domain (JM), represent clinically and biologically distinct entities and the most common types of *FLT3* mutation, as they occur in about 10% to 30% of AML cases [10,11]. *FLT3*-ITD mutations involve the insertion of a variable number of bases, resulting in a constitutively active aberrant protein and leading to uncontrolled proliferation of leukemic blasts, self-renewal, and resistance to differentiation and apoptosis signals [12]. ITD mutations are preferentially enriched within leukemia stem cells (LSCs) characterized by the CD34^+^/CD123^+^/CD25^+^/CD99^+^ immunophenotype. This signature identifies *FLT3*-ITD-positive LSCs with high specificity and supports the concept that these cells represent a reservoir of measurable residual disease (MRD) and a potential driver of disease recurrence [13]. Clinically, ITD mutations of *FLT3* significantly increase the risk of relapse, even in patients who initially achieve complete remission (CR), leading to chemotherapy resistance and decreased overall survival (OS) [14].

While 2017 European LeukemiaNet (ELN) guidelines recommended the use of allelic ratio (AR) values for risk stratification based on *NPM1* co-mutations [15], the updated 2022 ELN recommendations [16] substantially revised the classification of *FLT3*-ITD, categorizing AML as intermediate-risk regardless of AR and *NPM1* mutational status [8,17]. These guidelines reflect current treatment algorithms for *FLT3*-ITD mutated (*FLT3*-ITD^mut^) AML, emphasizing rapid *FLT3* mutation detection at diagnosis to enable early integration of *FLT3* inhibitors with intensive induction chemotherapy in fit patients. In the frontline setting, FLT3i include midostaurin, a first-generation multikinase inhibitor with activity against both *FLT3*-ITD and *FLT3*-TKD (tyrosine kinase domain) mutations, added to conventional chemotherapy as established by the RATIFY trial [18]. More recently, the type II *FLT3* inhibitor quizartinib used in the phase III QuANTUM-First study improved survival outcomes in patients with newly diagnosed *FLT3*-ITD^mut^ AML treated with 3 + 7 chemotherapy, followed by allogeneic stem-cell transplantation (allo-HSCT) in eligible patients [19]. In contrast, the FLT3i gilteritinib is approved as single-agent therapy in relapsed/refractory AML, based on the results of the ADMIRAL trial [20].

In this context, the concept of MRD has gained increasing importance as a tool to detect very low levels of leukemic cells persisting after therapy and ultimately driving disease progression or recurrence [21]. Across validated molecular and immunophenotypic MRD studies, MRD positivity after induction and consolidation, or before allo-HSCT, is consistently associated with an increased risk of relapse and inferior survival, leading to its incorporation into post-remission risk stratification algorithms [22,23,24]. *FLT3*-ITD mutations have been shown to occur at subclonal levels at diagnosis, and may be acquired or lost along disease progression or under therapeutic pressure, reflecting a high degree of clonal instability [9]. Moreover, *FLT3*-ITD mutations are highly patient-specific, displaying considerable heterogeneity in length, insertion site, and genomic sequence [25,26,27,28]. This behavior poses a major challenge for MRD assessment, as the clearance of *FLT3* mutations does not necessarily correspond to the eradication of the leukemia burden. Indeed, relapse may arise from ancestral *FLT3* wild-type clones or from newly emerging microclones that can persist below the detection threshold of conventional assays, thereby limiting the reliability of *FLT3* as a standalone MRD marker [29,30]. As a result of the biological complexity of ITD clones, 2022 ELN MRD recommendations [31] considered *FLT3*-ITD suboptimal for routine MRD monitoring when assessed using conventional next-generation sequencing (NGS), as compared to more stable molecular markers, such as *NPM1* mutations or recurrent fusion transcripts (e.g., RUNX1/RUNX1T1, CBFB/MYH11, PML/RARA, BCR/ABL1) which are recommended for MRD monitoring by real-time PCR (qRT-PCR) [32]. However, recent advances in ultra-high sensitivity (UHS)-NGS technologies have substantially challenged this paradigm, leading to the 2025 update of MRD guidelines in AML, by the ELN-DAVID MRD working party [21]. Indeed, dedicated UHS-NGS-based assays and specialized bioinformatic pipelines, such as the *getITD* protocol [33], have substantially improved detection and quantification of *FLT3*-ITD mutations, enabling the monitoring of patient-specific *FLT3*-ITD clones [21].

Our review addresses an unmet and highly relevant clinical need in the field of *FLT3*-ITD^mut^ AML: turning *FLT3*-ITD from a diagnostic/prognostic marker into a reliable MRD marker.

## 2. Biology and Clonal Dynamics of FLT3-ITD in AML

FLT3 is a member of the class III receptor tyrosine kinase (RTK) family, which also includes KIT, PDGFRα/β (platelet derived growth factor receptor alpha/beta), and CSF1R (colony stimulating factor 1 receptor) [34]. These receptors play essential roles in hematopoietic development, cellular proliferation, survival, and differentiation. Following post-translational processing, FLT3 is expressed as an immature intracellular isoform and a mature fully glycosylated form localized at the cell surface, where it mediates ligand-dependent signaling [35]. Structurally, FLT3 comprises an extracellular ligand-binding region, a transmembrane domain, an intracellular juxtamembrane domain (JMD), and a tyrosine kinase domain (TKD) [36].

*FLT3* mutations are detected in approximately 30% of newly diagnosed AML, with *FLT3*-ITD and *FLT3*-TKD point mutations representing the most common variants included in routine diagnostic assessment [37,38]. Less frequent non-canonical *FLT3* variants have also been reported in approximately 2% of AML cases, although their biological and clinical significance remains unclear [39,40]. Internal tandem duplications in the *FLT3* gene, which typically affect the cytoplasmic JM domain (exons 14–15), represent in-frame mutations induced by duplication of a variable number of bases (ranging from 3 to more than 400), all of which are multiples of three [12]. Therefore, the insertion, while not altering the normal reading frame, results in the translation of an abnormal protein that dimerizes independently of the ligand stimulation, promoting leukemic cell survival and impaired differentiation [41].

In contrast to more stable markers such as *NPM1*, *FLT3*-ITD are generally considered late events during leukemogenesis [42], often subclonal and unstable over time in up to 25% of AML patients [42,43,44]. Indeed, longitudinal analyses have shown that ITD mutational status may change over the course of disease, with the mutation either being lost or newly acquired at relapse. These dynamics likely reflect the expansion of pre-existing *FLT3*-ITD-mutated microclones that were below the limit of detection at diagnosis, or the persistence of clones that survive therapy and subsequently acquire additional genetic lesions [30]. Consistent with this clonal evolution model, approximately 15–30% of patients harboring *FLT3*-ITD at diagnosis relapse with *FLT3* wild-type disease, whereas a smaller subset of patients initially lacking *FLT3*-ITD acquire the mutation at relapse [9,45].

The introduction of FLT3i further highlighted the evolutionary plasticity of *FLT3*-ITD^mut^ AML. Correlative analyses from the RATIFY trial demonstrated that in approximately 46% of patients relapsing after midostaurin-based therapy *FLT3*-ITD mutations became undetectable at relapse, suggesting the expansion of *FLT3*-independent leukemic populations [8]. Likewise, molecular studies of gilteritinib-treated patients revealed the loss of *FLT3*-mutated clones in approximately 20% of cases, whereas resistant disease was associated with the emergence of *RAS/mitogen-activated protein kinase* (*MAPK*)-driven subclones or secondary *FLT3* kinase-domain mutations [46]. In contrast, resistance to quizartinib more commonly involves on-target evolution through the acquisition of *FLT3*-TKD mutations, particularly affecting D835 and F691 residues [47]. These observations indicate that relapse after FLT3i therapy frequently reflects clonal remodeling rather than persistence of the dominant diagnostic clone.

Altogether, these findings establish *FLT3*-ITD as a highly dynamic molecular lesion whose behavior mirrors the evolutionary trajectory of AML. In the era of *FLT3* targeted therapies, understanding this clonal complexity is essential for interpreting molecular response and mechanisms of resistance, and underscores the challenges of using *FLT3*-ITD as a reliable MRD marker.

## 3. Methodologies for FLT3-ITD MRD Detection and the Updated 2025 ELN MRD Guidelines

Despite genetic profiling having improved the definition of prognostic risk in AML, disease relapse remains the leading cause of treatment failure. Although available treatments induce morphologic complete remission in about 80% of AML patients, 30–40% of them eventually relapse due to the survival of leukemic cells resistant to conventional therapies, which are capable of re-expanding and causing disease relapse [48,49,50]. In this setting, MRD assessment is a cornerstone of AML management, providing prognostic information beyond conventional morphology and enabling the identification of patients at increased risk of relapse [51]. The most recent ELN-DAVID 2025 international guidelines [21] have further strengthened the clinical relevance of MRD monitoring by integrating molecular subtype, treatment phase, and disease kinetics into risk-adapted treatment planning.

Before the introduction of high-sensitivity sequencing technologies, MRD monitoring employed multiparameter flow cytometry (MFC) and molecular assays targeting recurrent genetic abnormalities. Although not particularly sensitive, flow cytometry can be used in the majority of AML patients (>90%), contributing to risk stratification at different stages of treatment. MFC-based MRD assessment relies on the identification of a leukemia-associated immunophenotype (LAIP), defined as an antigen expression pattern characteristic of myeloid blasts and informative during patients’ follow-up [52,53]. In the absence of a suitable LAIP, the ELN 2025 guidelines endorse the “different from normal” (DfN) approach, which identifies immunophenotypic deviations from normal hematopoietic maturation [21].

qRT-PCR remains the reference method for molecular MRD assessment whenever a validated molecular target is available. Among these, *NPM1* mutations represent the most extensively validated target because of their biological stability and close correlation with leukemic burden [22,54,55].

**Figure 1:**
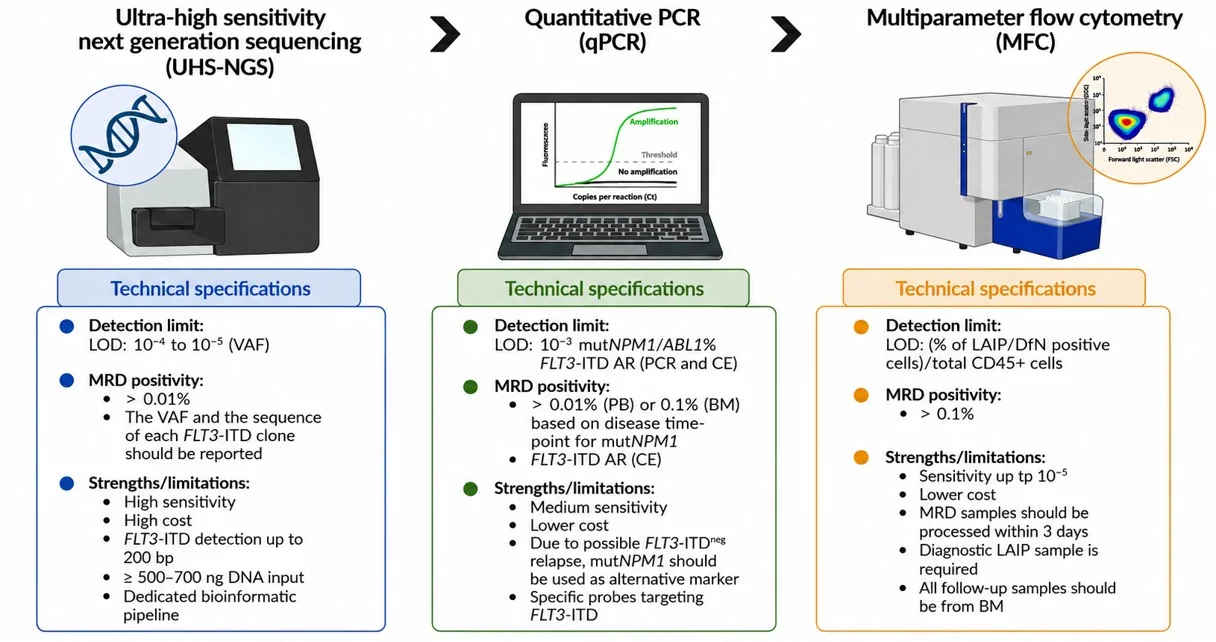
Comparison of MRD detection methods in *FLT3*-ITD^mut^ AML. The panels highlight the main features of the three principal methods currently employed for MRD assessment in *FLT3*-ITDmut AML, emphasizing their complementary roles in MRD monitoring and risk stratification in AML. MRD: measurable residual disease; UHS-NGS: ultra-high sensitivity next generation sequencing; qPCR: quantitative PCR; MFC: multiparameter flow cytometry; LOD: limit of detection; AR: allelic ratio; VAF: variant allele frequency; PB: peripheral blood; BM: bone marrow; CE: capillary electrophoresis; LAIP: leukemia-associated immunophenotype; DfN: different from normal. Created with BioRender.com.

In the context of *FLT3*-ITD^mut^ AML, MRD monitoring represents a challenging and constantly evolving field, in which conventional and more innovative techniques coexist, albeit with different limitations and potential (Figure 1) [56].

Historically, *FLT3*-ITD detection relied on DNA fragment analysis by capillary electrophoresis (CE) [57], which, although highly effective and routinely used for diagnostic purposes, exhibits limited analytical sensitivity (approximately 10^−1^–10^−2^) and is prone to amplification bias due to preferential amplification of shorter fragments. The limited sensitivity of this traditional diagnostic approach has long hindered the detection of low-level *FLT3*-ITD-positive clones, contributing to the underestimation of its persistence over time, especially in a context characterized by high molecular heterogeneity. As a result, residual *FLT3*-ITD detection has traditionally been considered of limited prognostic value, particularly when compared with more stable leukemic mutations [21]. The well-known intrinsic complexity of ITD variants and, above all, the historical lack of standardization across laboratories also impaired the comparison of results across different trials.

**Figure 2:**
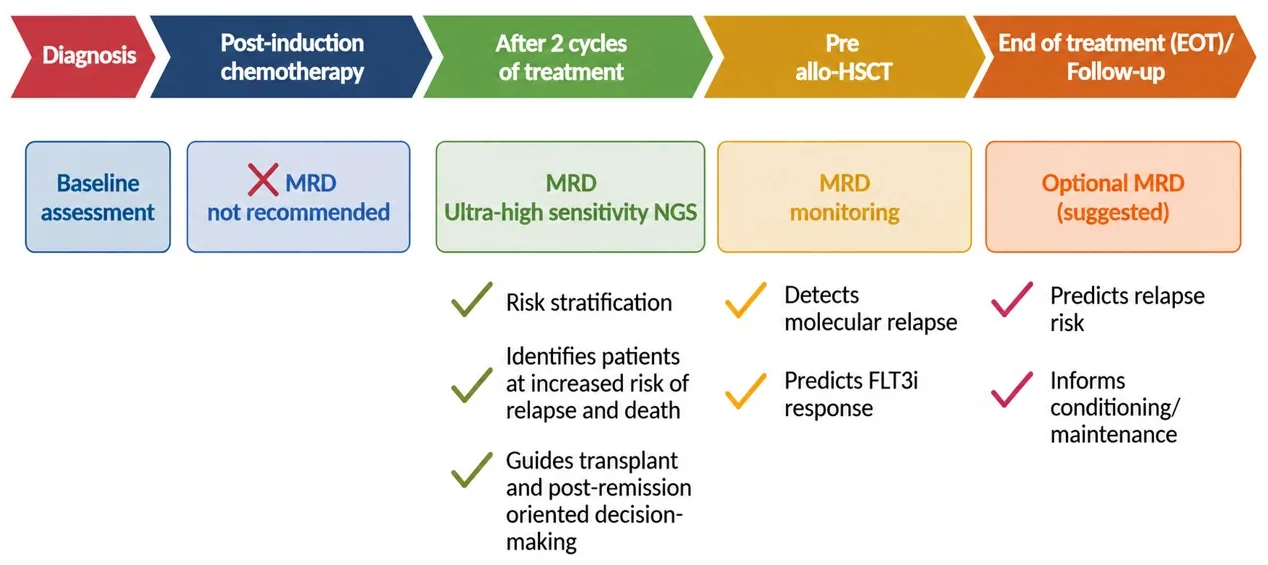
Timeline of MRD assessment in *FLT3*-ITD^mut^ AML. Overview of the informative time-points for the detection of *FLT3*-ITD mutational burden. Check marks (✓) indicate the clinical applications/implications of MRD assessment at the corresponding time points, whereas the cross mark (✕) indicates that MRD assessment is not recommended. MRD: measurable residual disease; NGS: next-generation sequencing; HSCT: hematopoietic stem cell transplantation; FLT3i: FLT3 inhibitors. Created with BioRender.com.

The ELN-DAVID 2025 recommendations have therefore introduced several key changes to improve the accuracy and clinical role of *FLT3*-ITD MRD assessment in AML. Among these, particular emphasis has been placed on the recommendation to use UHS-NGS methods for the detection of the *FLT3*-ITD mutational burden after two cycles of chemotherapy and prior to allo-HSCT, as these time-points provide the strongest prognostic information. Although assessment at the end of treatment (EOT) and during follow-up may also be informative, supporting evidence remains less compelling (Figure 2) [21,58,59].

From a technical point of view, *FLT3*-ITD sequencing can be performed using either a single-step or a two-step PCR-based NGS, with specific primers amplifying exons 14–15 [60,61,62]. The targeted deep sequencing technique enabled a significant improvement in analytical sensitivity, achieving a limit of detection (LOD) as low as 10^−4^–10^−5^, thus allowing the identification of very low-frequency residual clones. Specifically, it has been established that the cut-off for MRD positivity for *FLT3*-ITD must correspond to a LOD of at least 0.01% [21]. Despite its extreme sensitivity in detecting mutations, MRD assay by UHS-NGS is largely affected by the size and the DNA amount of samples [24,63]. Actually, it detects up to 228 bp *FLT3*-ITDs [64] and requires at least 500 ng DNA input (700 ng for increased confidence in detecting false negatives), a minimum number of identical reads to establish positivity, and a dedicated bioinformatics pipeline to analyze the results, which is crucial for the correct data interpretation [21]. A major advance is represented by the introduction of the *getITD* algorithm [33], which enabled standardized NGS-based *FLT3*-ITD detection, improving accuracy and sensitivity, and facilitated subsequent clinical investigations of *FLT3*-ITD MRD. In order to ascertain the extent of the disease in patients presenting with multiple ITD clones, reporting the VAF values of all individual ITD clones identified, along with their sequences, is required. Furthermore, although it has been established that the major clone reflects the level of MRD, providing the total VAF, calculated by summing the individual VAFs of the different clones, is also strongly recommended [21]. Although the UHS-NGS method allows for the detection of clones with very low VAF, in cases of AML relapses that are negative for the *FLT3*-ITD mutation, it is necessary to rely on conventional MRD methods at each FU time-point. Indeed, as pointed out several times, a patient may have a *FLT3*-ITD negative relapse, or *FLT3*-ITD mutation may drive disease relapse. The expert panel further suggests performing these analyses preferentially on bone marrow (BM) rather than peripheral blood (PB) samples, as it is well known that MRD levels are higher in BM [21].

Summing up, while the biological stability of the *NPM1* mutation makes it a reliable marker reflecting the leukemic burden, the subclonal and dynamic nature of *FLT3*-ITD requires an integrated approach that considers the timing of testing, the clonal history of the disease, and current therapeutic strategies, including exposure to FLT3 inhibitors. Thus, *FLT3*-ITD helps refine risk stratification, particularly at key stages of the therapeutic path, as in the pre-transplant phase.

## 4. Clinical Evidence of FLT3-ITD MRD

Measurable residual disease is crucial information for allo-HSCT allocation [22]. In this setting, *FLT3*-ITD detection represents a challenging application of NGS, as these structural variants exhibit highly variable sizes and generate complex alignment patterns that are not optimally handled by standard variant-calling pipelines. However, some of these technical caveats have been overcome by updated NGS-based assays [65,66]. Indeed, recent data showed that *FLT3*-ITD microclones, detectable only by UHS-NGS approaches, are independently associated with an increased risk of relapse in AML patients [67,68]. Interestingly, using an NGS-based analysis and a panel covering the entire *FLT3* coding region, Duployez et al. showed that approximately 17% of patients (*n* = 1380) who tested negative (mutation not detectable or with an AR below the threshold) for ITD mutation by fragment analysis had at least one microclone, with a median AR of 3.9 × 10^−3^. Moreover, authors demonstrated that microclones, albeit found at very low levels at diagnosis, were clinically relevant and were associated with an increased incidence of relapse compared with ITD-negative cases (*p* = 0.001), whereas there were no significant differences in terms of CR following intensive chemotherapy, even when compared to patients presenting with macroclones (AR ≥ 0.05). Of note, 2- and 5-year cumulative incidence of relapse (CIR) rates were higher in patients with *FLT3*-ITD microclones (45% and 48%) compared with other patient groups. Similarly, microclones exhibited a clear impact on relapse-free survival (RFS), but their effect on OS was less pronounced and did not reach statistical significance. Of particular interest, the analysis of 306 paired diagnosis-relapse samples also showed that, in most cases, *FLT3*-ITD microclones present at diagnosis may expand and become the dominant clone at relapse, suggesting that they act as a reservoir of residual disease capable of spreading and causing relapse. Indeed, in nearly 40% of AML cases treated with intensive chemotherapy without midostaurin (23 out of 55 patients), the *FLT3*-ITD^mut^ clone at relapse matched one of the microclones already present at diagnosis, particularly in cases with higher baseline AR, reflecting a pattern of clonal selection.

Grob et al. reported the results from a cohort of 161 *FLT3*-ITD AML, showing that 29% of patients are *FLT3*-ITD+ after two cycles of induction chemotherapy despite achieving morphological remission, and that *FLT3*-ITD^mut^ was associated with an increased risk of relapse and reduced OS [69]. Specifically, NGS analysis revealed more than double the incidence of relapse at 4 years in patients with *FLT3*-ITD^mut^ AML who had detectable MRD (75% vs. 33%, *p* < 0.001). Correspondingly, OS was around 30% in the *FLT3*-ITD MRD+ group vs. 57% among the 114 patients who were *FLT3*-ITD MRD- (*p* < 0.001). Notably, MRD clones were identical to those at diagnosis and their size correlated with the recurrence rate. In addition to *FLT3*-ITD+, only white blood cell count (WBC) and late CR were associated with an increased risk of relapse and reduced OS, but not the presence of *NPM1* mutation and *FLT3*-ITD AR. Similarly, a recent study by Lee and colleagues demonstrated the feasibility of highly sensitive longitudinal monitoring of *FLT3*-ITD clones by NGS-based assays, providing the technical foundation for the clinical validation of *FLT3*-ITD MRD as a prognostic and potentially predictive biomarker in AML [70].

Overall, detection of *FLT3*-ITD MRD, assessed using highly sensitive NGS technologies, identifies a subset of patients at high risk of treatment failure. The implications for clinical practice are significant, as they suggest the need to integrate MRD assessment into clinical decision processes to select patients who are candidates for more intensive strategies, such as transplantation and/or maintenance with FLT3i. However, it is important to distinguish the well-established prognostic value of *FLT3*-ITD MRD from its potential predictive value. Current evidence consistently demonstrates that detectable *FLT3* MRD is associated with an increased risk of relapse and inferior survival outcomes across multiple treatment settings [22,58,65]. By contrast, whether *FLT3*-ITD MRD can reliably identify patients who will benefit from specific therapeutic interventions and can be routinely incorporated into treatment decision-making remains to be established in prospective clinical trials [21].

## 5. FLT3-ITD MRD and FLT3 Inhibitor-Based Therapies

Several clinical trials have proved the role of *FLT3* inhibitors at different treatment phases (Table 1), reshaping the therapeutic landscape of *FLT3*-ITD^mut^ AML and introducing new dimensions to MRD-guided management [16,22].

To date, FLT3i are commonly classified into first-generation inhibitors, including midostaurin (class I inhibitor) and sorafenib (class II inhibitor), and more potent and selective second-generation inhibitors, including gilteritinib (class I inhibitor) and quizartinib (class II inhibitor) [76,77]. Midostaurin was the first approved FLT3i and is indicated, in combination with standard 3 + 7 chemotherapy, for adults with newly diagnosed *FLT3*^mut^ AML, based on the results of the phase III RATIFY trial. In the European Union, the approved indication also includes single-agent maintenance in patients who achieve a complete response [8,18,71]. Sorafenib is a multikinase inhibitor primarily used as post-allo-HSCT maintenance therapy in *FLT3*-ITD^mut^ AML, based on evidence demonstrating a reduction in relapse rate. It is administered with off-label use and should be considered according to the clinical context and current guideline recommendations [72,78]. Gilteritinib is approved as monotherapy in relapsed or refractory *FLT3*^mut^ AML, following the survival benefit over salvage chemotherapy demonstrated in the phase III ADMIRAL trial [20]. Owing to its high selectivity for *FLT3*-ITD, quizartinib has been established as a therapeutic alternative to midostaurin when administered in association with induction and consolidation chemotherapy in patients with *FLT3*-ITD^mut^ AML, followed by single-agent maintenance therapy, based on the phase III QuANTUM-First trial [19]. In the phase III QUANTUM-R trial, quizartinib monotherapy demonstrated a significant improvement in overall survival compared with salvage chemotherapy in patients with relapsed or refractory *FLT3*-ITD^mut^ AML [73].

The impact of FLT3 inhibitors on MRD kinetics is heterogeneous and often fails to result in prolonged suppression of the leukemic clone. The principal clinical studies evaluating NGS-based *FLT3*-ITD MRD across different FLT3 inhibitor-based treatment strategies are summarized in Table 2.

In the AMLSG 16-10 trial, Rücker and colleagues emphasized the importance of using NGS-based assays to evaluate the mutational burden of *FLT3*-ITD MRD in a cohort of patients treated with intensive chemotherapy plus midostaurin, followed by midostaurin as maintenance therapy [79]. This study enrolled 157 *FLT3*-ITD^mut^ AML patients for whom BM samples were available at three distinct time-points: after two cycles of intensive chemotherapy (Cy2, *n* = 142), at the end of treatment (EOT, *n* = 116), and during follow-up (FU, *n* = 148). This controlled environment ensured the strength of the data obtained, combined with use of ultra-sensitive sequencing techniques, according to *getITD* protocol. Interestingly, prior to investigating the prognostic impact of *FLT3*-ITD MRD, the authors compared VAF values in paired BM and PB samples and demonstrated that, whereas there are no significant differences at the time of diagnosis, they increase after Cy2 (*p* < 0.001), underscoring the need to use BM as the preferred source for molecular analyses. Moreover, using NGS-based assessment at diagnosis, they identified 465 ITDs in the AML population, with a median VAF of 0.312%, underlining the effectiveness of this method in detecting mutations with very low VAF. They found that persistence of MRD positivity could help identify candidates for more aggressive treatment strategies, such as HSCT in first remission, or for more intensive therapy, including the use of FLT3i. Of note, pre-transplant MRD positivity has consistently been associated with a significantly higher risk of post-transplant relapse and reduced survival. Accordingly, Rücker et al. proved that 15 out of 21 patients with *FLT3*-ITD MRD+ before HSCT cleared ITD mutations after transplantation [79]. Consequently, MRD status is increasingly being investigated as an adjunctive tool to refine transplant risk assessment and optimize treatment strategies, although its role in guiding transplant decisions has not yet been prospectively validated. In some cases, patients with persistent MRD may benefit from additional therapy aimed at achieving deeper remission prior to transplantation.

Randomized studies demonstrated that the addition of sorafenib to chemotherapy [80] or its use as post-HSCT maintenance significantly reduced the risk of relapse, with a particularly pronounced effect in patients with MRD positivity after allogeneic transplantation [72,81]. In particular, in the SORMAIN trial 83 patients (median age 54 years) were randomly assigned to receive either sorafenib or placebo over a period of 60 to 100 days following HSCT [72]. Two years of maintenance therapy with sorafenib resulted in a substantially reduced rate of relapse in patients in CR (85% of RFS in the sorafenib group vs. 53.3% in the placebo group), suggesting that prolonged inhibition of *FLT3* may help manage residual disease, despite not fully eliminating it. Likewise, 24 months OS was significantly higher in the sorafenib arm (90.5% vs. 66.2% for placebo). The authors also assessed MRD status pre- and post-HSCT and underlined the benefit of adding sorafenib in patients who were MRD- prior to transplantation (*p* = 0.028) and in patients with post-HSCT MRD+ (*p* = 0.015). Despite its benefits, the clinical use of sorafenib is limited by off-target toxicities, frequent dose modifications and treatment discontinuations in real-world practice. Indeed, the study documented a higher number of cases of drug-related toxicity compared to the control group (22% vs. 5%), with a higher incidence of infections (26% vs. 23%) and graft-versus-host disease (GvHD) in the sorafenib group (77%), whereas the incidence of gastrointestinal toxicity was comparable between the two arms of the trial.

The ADMIRAL trial demonstrated that single-agent gilteritinib improves OS in patients with relapsed/refractory *FLT3*-ITD^mut^ AML compared to standard chemotherapy, but it also highlighted that the median duration of response is 4.9 months, with frequent relapses, suggesting that *FLT3* inhibition alone is not sufficient to eradicate residual disease [20,82]. Conversely, the use of FLT3i in the maintenance setting, particularly after HSCT, represents a promising strategy for suppressing residual disease and preventing relapse [74]. Data from the MORPHO trial, which included patients who achieved CR following transplantation, randomly assigned to receive gilteritinib or a placebo, revealed that maintenance therapy may prolong remission duration [74] with 77% RFS vs. 70% in the case of the placebo (cumulative incidence, CI 62.4 to 76.2), whereas OS was similar for the two arms. The RFS benefit was significant in MRD+ patients, whether assessed before or after transplantation [74,75]. Overall, this study showed that gilteritinib represents a promising strategy for post-HSCT maintenance therapy in patients with detectable MRD, whereas no statistically significant benefit was observed among MRD- patients, suggesting that MRD status should be used to select eligible patients for treatment [74]. However, because these findings derive from prespecified subgroup analyses of a trial that did not meet its primary endpoint, they should not be interpreted as evidence that MRD- patients derive no benefit from gilteritinib. Rather, they support further prospective evaluation of MRD-guided maintenance strategies.

The QuANTUM-First trial showed that adding quizartinib to intensive first-line chemotherapy improved survival in *FLT3*-ITD^mut^ AML, but failed to overcome the persistence of MRD, which still predicts relapse in this setting [58]. MRD status was evaluated at specific time-points (after induction, after consolidation before maintenance and pre-HSCT) in a cohort of patients, comprising both those achieving CR and CR with partial hematologic recovery (CRi), defining the subset of composite CR (CRc) [58]. In detail, the analysis showed that, among the 368 patients in CRc and the 297 patients in CR for whom MRD samples were available, those who were assigned to the quizartinib arm experienced an increased rate and depth of *FLT3*-ITD clearance compared to the placebo. This finding was observed during the first and/or second induction cycle following administration of quizartinib, with a reduction in the *FLT3*-ITD VAF, by 2.8-fold (*p* = 0.0439) and 8-fold (*p* = 0.1345), respectively. A similar trend applied when considering the presence of the *NPM1* co-mutation. Indeed, unlike previous studies in which *NPM1* mutation was considered a *condicio sine qua non* for MRD negativity [83], the present study demonstrates that quizartinib can induce deep remission regardless of the presence of *NPM1* co-mutation in patients with CRc [58]. Importantly, as suggested by the most recent guidelines [21], the authors addressed the importance of choosing a specific cut-off value to achieve RFS, as the absence of detectable mutant alleles below a predefined sensitivity threshold was strongly associated with improved clinical outcomes. Indeed, the use of a 10^−4^ cut-off in patients in CRc and CR after induction including quizartinib was associated with improved RFS (hazard ratio, HR: 0.791), whereas no significant differences in the choice of cut-offs (10^−4^ vs. 0) were observed if the measurement was performed prior to maintenance therapy. Similarly, treatment with quizartinib was effective in converting MRD status from positive to negative after consolidation or maintenance, regardless of transplantation. Furthermore, the addition of the FLT3i conferred a survival advantage in patients undergoing transplantation in CR1, but not in CRc1, irrespective of pre-transplant MRD status [58].

Although these data support a dynamic model in which MRD not only represents a prognostic marker, but is also a guide to the use of *FLT3* inhibitors along the therapeutic continuum, they remain heterogeneous and several methodological and clinical issues still limit its routine implementation. The major clinical trials evaluating FLT3i were primarily designed to assess therapeutic efficacy rather than to prospectively validate *FLT3*-ITD MRD as a biomarker. Consequently, the evidence supporting MRD-guided therapeutic decisions is largely derived from secondary or exploratory analyses. Accordingly, prospective interventional studies demonstrating that MRD-directed therapeutic strategies improve survival are still lacking. Therefore, *FLT3* MRD should currently be regarded as a valuable prognostic tool that refines risk stratification and supports clinical decision-making, rather than a standalone biomarker sufficient to guide treatment selection in routine clinical practice.

## 6. Integration of FLT3-ITD MRD into Clinical Decision-Making

Post-induction and peri-HSCT *FLT3*-ITD MRD, tested via UHS-NGS (able to achieve sensitivity <0.1%), has been recently implemented in the ELN25 MRD guidelines [21]. However, as previously described, several lines of evidence correlated *FLT3*-ITD MRD status with clinical outcomes [64,69,75] and few studies, leveraging different molecular targeting for monitoring, explored the possibility of molecular MRD-directed decision process. In particular, the analysis of NCRI (National Cancer Research Institute) AML17 and AML19 phase III trials showed that in patients harboring both *NPM1* mutations and *FLT3*-ITD, MRD monitoring (using established molecular biomarkers including *NPM1* mutations, core binding factor translocations or *KMT2A* rearrangements) was associated with a significant reduction in mortality (3-year OS: 69% vs. 58%, HR 0.53). In particular, the early identification of molecular relapse allowed for a pre-emptive treatment in 45% of MRD+ patients [84]. Similarly, the Italian GIMEMA AML1310 trial incorporated MRD assessment through both PCR (in *NPM1*-mutated and core-binding factor AML) and MFC in the remaining cases for decision-making [85]. Patients were stratified according to National Comprehensive Cancer Network (NCCN) risk categories and MRD status, and assigned to allogenic or autologous HSCT. In the overall cohort, 2-year OS and relapse-free survival (RFS) were 56% and 54%, respectively. Notably, 2-year OS and disease-free survival (DFS) were 74% and 61% in the favorable-risk group, 42% and 45% in the poor-risk group, 79% and 61% in the intermediate-risk MRD-negative group, and 70% and 67% in the intermediate-risk MRD-positive group. Importantly, outcomes in MRD-positive intermediate-risk patients undergoing allogeneic HSCT were comparable to those observed in the favorable-risk category, supporting the clinical benefit of MRD-driven allocation to transplant in this subgroup.

These papers emphasize the key importance of pre-HSCT MRD monitoring since its positivity, irrespective of the specific marker used, identifies patients at high risk of relapse and inferior outcome, and should prompt consideration of pre-emptive or intensified therapeutic strategies, including the use of FLT3 inhibitors in patients with a known *FLT3*-mutated disease history [86,87].

## 7. Conclusions and Future Directions

The increasing body of evidence supporting *FLT3*-ITD MRD assessment has underscored its potential as a key biomarker for refining risk stratification in *FLT3*-ITD^mut^ AML, particularly with the advent of *FLT3* targeted therapies [58,69,72,74,79,82]. Despite these advances, several issues remain to be addressed before *FLT3*-ITD MRD can be routinely implemented in clinical practice and integrated into prospective therapeutic algorithms. A critical step toward clinical implementation is the standardization of laboratory methodologies. To date, considerable heterogeneity in NGS platforms, amplification strategies, bioinformatic pipelines, and reporting practices has limited the comparability of findings across studies. Therefore, harmonization of pre-analytical procedures, sequencing strategies, data analysis pipelines, and MRD reporting standards will be critical to ensuring the reproducibility and comparability of results across institutions [21,33,69]. Beyond technical considerations, important biological uncertainties also remain. *FLT3*-ITD frequently represents a secondary or subclonal genetic event and may be lost or newly acquired during disease evolution, thereby complicating the interpretation of MRD results [21,22]. In addition, the selective pressure exerted by FLT3i may further influence clonal dynamics, contributing to changes in *FLT3*-ITD status over the course of the disease [9,43,46]. Consequently, discordant MRD results may occur between *FLT3*-ITD and established molecular biomarkers, particularly *NPM1*, which currently represents the benchmark for molecular response assessment, reflecting differences in clonal architecture, disease biology, and assay sensitivity [22,49]. It is indeed well recognized that molecular clearance of *FLT3*-ITD mutations is generally less deep and less durable than that observed for other MRD markers, highlighting the lack of adequate evidence to support systematic prioritization of one molecular marker over another for treatment decision-making when inconsistent findings are observed [9]. MRD results should be interpreted within the overall biological and clinical context, considering baseline molecular characteristics, disease genetics, treatment history, and longitudinal MRD kinetics. Accordingly, *FLT3*-ITD MRD should be regarded as complementary to, rather than a replacement for, validated molecular MRD markers. Such an integrated approach may be particularly valuable in *NPM1* wild-type AML and in patients receiving FLT3i-based therapies.

The optimal source for *FLT3*-ITD MRD monitoring also requires further investigation. Although BM is currently considered the preferred specimen due to its higher analytical sensitivity, recent studies have reported a good concordance between BM and PB MRD assessments, suggesting that PB may represent a feasible alternative for serial monitoring in selected clinical settings [58,79]. The potential implications of using PB as a substitute for BM sampling are yet to be validated.

On a clinical level, the most important future application of *FLT3*-ITD MRD lies in the development of MRD-guided therapeutic strategies. Indeed, MRD not only represents a powerful prognostic factor but also serves as an essential tool to identify patients who may derive the greatest benefit from allo-HSCT, post-transplant maintenance, or pre-emptive FLT3i therapy at molecular relapse [21]. Further methodological standardization, biological validation, and prospective clinical studies are, however, required before *FLT3*-ITD MRD predictive value and routine integration into MRD-guided therapeutic decision-making can be established.

## Figures and Tables

**Table 1:** Major clinical studies of FLT3 inhibitors in AML.

Clinical Study	Target Population	FLT3 Inhibitor	Treatment Backbone	Principal Clinical Outcome
RATIFY (CALGB 10603) [18]	Newly diagnosed AML with *FLT3* mutation (ITD or TKD)	Midostaurin	Standard induction with daunorubicin/cytarabine and high-dose cytarabine consolidation plus midostaurin or placebo; protocol-defined maintenance with the assigned agent; allo-HSCT permitted	Significantly improved OS and EFS with midostaurin
AMLSG 16-10 [71]	Newly diagnosed AML with *FLT3*-ITD mutation	Midostaurin	Midostaurin combined with intensive chemotherapy, followed by allo-HSCT when indicated and 1 year of single-agent midostaurin maintenance	Improved EFS and OS compared with controls
SORMAIN [72]	AML with *FLT3*-ITD mutation in CR after allo-HSCT	Sorafenib	Sorafenib or placebo for up to 24 months as post-transplant maintenance	Significantly improved RFS; reduced risk of relapse or death
ADMIRAL [20]	Relapsed/refractory AML with *FLT3* mutation (ITD or TKD D835/I836)	Gilteritinib	Single-agent gilteritinib vs. investigator-selected salvage chemotherapy	Significantly improved OS and CR/CRh rate
QuANTUM-R [73]	Relapsed/refractory AML with *FLT3*-ITD mutation	Quizartinib	Single-agent quizartinib vs. investigator-selected salvage chemotherapy	Significantly improved OS
QuANTUM-First [19]	Newly diagnosed AML with *FLT3*-ITD mutation	Quizartinib	Standard induction and consolidation chemotherapy plus quizartinib or placebo, followed by continuation therapy with the assigned agent	Significantly prolonged OS with quizartinib
MORPHO (BMT CTN 1506) [74,75]	AML with *FLT3*-ITD mutation in CR1 undergoing allo-HSCT	Gilteritinib	Gilteritinib or placebo for 24 months as post-transplant maintenance	The primary RFS endpoint was not statistically significant in the overall population; RFS benefit was observed in patients with detectable peri-HSCT *FLT3*-ITD MRD

**Abbreviations:** AML: acute myeloid leukemia; CR: complete remission; CR1: first complete remission; CRh: complete remission with partial hematologic recovery; EFS: event-free survival; HSCT: hematopoietic stem-cell transplantation; ITD: internal tandem duplication; MRD: measurable residual disease; OS: overall survival; RFS: relapse-free survival; TKD: tyrosine kinase domain.

**Table 2:** Principal clinical studies supporting NGS-based FLT3-ITD MRD in AML.

Clinical Study	Target Population	FLT3 Inhibitor	Treatment Backbone	Specimen	*FLT3*-ITD MRD Assay	Analytical Sensitivity	Clinical MRD Positivity Threshold	Sampling Time Point(s)	Clinical Outcome	Key Limitations
HOVON-SAKK [69]	Newly diagnosed *FLT3*-ITD AML in CR	None (pre-FLT3 inhibitor era)	Intensive chemotherapy	BM	Deep NGS targeting exon 14	10^−5^	≥10^−5^	Post-induction CR	Independent predictor of relapse and OS	Therapeutically heterogeneous cohort
AMLSG 16-10 [79]	Newly diagnosed *FLT3*-ITD AML	Midostaurin	Intensive chemotherapy + maintenance	BM/PB	Paired-end NGS + *getITD* protocol for bioinformatic analysis	10^−5^	≥10^−5^	After two cycles of intensive chemotherapy; EOT; follow-up	Strong predictor of EFS and OS; conversion to positivity anticipated relapse	Single-arm study; lack of a standardized NGS-based *FLT3*-ITD MRD assay
MORPHO [74]	*FLT3*-ITD AML after allo-HSCT	Gilteritinib	Maintenance post-HSCT vs. placebo	BM	PCR-NGS targeting exons 14–15	10^−6^	Detectable MRD	Peri-HSCT	Benefit in terms of RFS and OS mainly in MRD+ patients	MRD subgroup exploratory
QuANTUM-First [58]	Newly diagnosed *FLT3*-ITD AML	Quizartinib	Intensive chemotherapy + maintenance	BM	UHS-NGS targeting exons 14–15	2 × 10^−6^	≥10^−4^	After induction; after consolidation before maintenance; before allo-HSCT	Significant improvement of RFS and OS; deeper *FLT3*-ITD clearance was associated with better outcomes	Lack of MRD samples in consolidation or maintenance; lack of a standardized *FLT3*-ITD MRD assay; exploratory analysis
Pre-MEASURE [64]	*FLT3*-ITD AML in CR1 prior to allo-HSCT	Not applicable	Allo-HSCT	PB	PCR-NGS	10^−6^	≥10^−4^	Pre-HSCT	Strong predictor of relapse and OS; persistent pre-HSCT *FLT3*-ITD was associated with increased relapse and mortality	Therapeutically heterogeneous cohort and MRD assessment restricted to the pre-transplant setting
UK–Australia retrospective cohort [65]	*FLT3*-ITD AML underwent allo-HSCT	Different FLT3i	Intensive chemotherapy w/ or w/o FLT3i	BM/PB	PCR-NGS targeting exons 14–15	10^−5^	≥10^−5^	Pre-HSCT	Even very low pre-HSCT *FLT3*-ITD levels predicted relapse and inferior survival	Therapeutically heterogeneous cohort

**Abbreviations:** AML: acute myeloid leukemia; BM: bone marrow; CR: complete remission; EFS: event-free survival; FLT3i: FLT3 inhibitor; *FLT3*-ITD: *fms related receptor tyrosine kinase 3*-internal tandem duplication; HSCT: hematopoietic stem-cell transplantation; MRD: measurable residual disease; OS: overall survival; PB: peripheral blood; RFS: relapse-free survival; UHS-NGS: ultra-high sensitivity next-generation sequencing.

## Data Availability

No new data were created or analyzed in this study. Data sharing is not applicable to this article.

## Abbreviations

The following abbreviations are used in this manuscript:

AML	Acute myeloid leukemia
AR	Allelic ratio
BM	Bone marrow
CE	Capillary electrophoresis
CIR	Cumulative incidence of relapse
CR	Complete remission
CRc	Composite complete remission
CRi	Complete remission with partial hematologic recovery
CSF1R	Colony stimulating factor 1 receptor
DfN	Different from normal
DFS	Disease-free survival
EBMT	European Society for Blood and Marrow Transplantation
ELN	European LeukemiaNet
EOT	End of treatment
FLT3	Fms-related receptor tyrosine kinase 3
FLT3i	FLT3 inhibitors
FU	Follow-up
GIMEMA	Gruppo Italiano Malattie EMatologiche dell’Adulto
GvHD	Graft-versus-host disease
HSCT	Hematopoietic stem cell transplantation
ITD	Internal tandem duplication
JM	Juxtamembrane domain
LAIP	Leukemia associated immunophenotype
LOD	Limit of detection
LSCs	Leukemia stem cells
MAPK	Mitogen-activated protein kinase
MFC	Multiparameter flow cytometry
MRD	Measurable residual disease
NCCN	National Comprehensive Cancer Network
NCRI	National Cancer Research Institute
NGS	Next-generation sequencing
NPM1	Nucleophosmin
OS	Overall survival
PB	Peripheral blood
PDGFRα/β	Platelet-derived growth factor receptor alpha/beta
qRT-PCR	Quantitative Reverse Transcription-Polymerase Chain Reaction
RFS	Relapse-free survival
RTK	Receptor tyrosine kinase
TKD	Tyrosine kinase domain
TKIs	Tyrosine kinase inhibitors
UHS	Ultra-high sensitivity
VAF	Variant allele frequency
WBC	White blood cell count
